# Risk of Incident Post-Transplantation Diabetes Mellitus After Solid Organ Transplantation in Taiwan: A Population-Based Cohort Study

**DOI:** 10.3390/healthcare13050523

**Published:** 2025-02-27

**Authors:** Chih-Jaan Tai, Kuang-Hua Huang, Jiun-Yi Wang, Shuo-Yan Gau, Shiang-Wen Huang, Kun-Yu Su, Tung-Han Tsai, Chun-Nan Wu, Chien-Ying Lee

**Affiliations:** 1 Department of Otorhinolaryngology-Head and Neck Surgery, China Medical University Hospital, Taichung 404327, Taiwan; edsam@seed.net.tw; 2 School of Medicine, China Medical University, Taichung 404328, Taiwan; 3 Department of Health Services Administration, China Medical University, Taichung 406040, Taiwan; khhuang@mail.cmu.edu.tw (K.-H.H.); dondon0525@gmail.com (T.-H.T.); 4 Department of Healthcare Administration, Asia University, Taichung 413305, Taiwan; jjwang@asia.edu.tw; 5 Department of Medical Research, China Medical University Hospital, Taichung 404327, Taiwan; 6 School of Medicine, Chung Shan Medical University, Taichung 40201, Taiwan; sixsamurai.shien15@gmail.com (S.-Y.G.); aprilhuangsw520@gmail.com (S.-W.H.); s1001024@gm.csmu.edu.tw (K.-Y.S.); 7 Department of Business Administration, National Taiwan University, Taipei 106319, Taiwan; 8 Department of Pharmacology, Chung Shan Medical University, Taichung 40201, Taiwan; 9 Department of Pharmacy, Chung Shan Medical University Hospital, Taichung 40201, Taiwan

**Keywords:** posttransplant diabetes mellitus, solid organ transplant, cohort study, real-world data

## Abstract

**Background:** Solid organ transplant (SOT) recipients have an elevated risk of diabetes mellitus (DM). This study investigated the risk of posttransplant DM (PTDM) in a retrospective cohort study. **Methods:** We analyzed patients aged over 18 years who received an SOT between 2002 and 2013. Each patient was matched with four control individuals by age, sex, insured salary, urbanization level, Charlson’s comorbidity index (CCI), and year of inclusion in the study. After matching, the study comprised 6874 patients who underwent an SOT and 27,496 matched general patients as the comparison. The risk of DM among the SOT recipients was assessed using a Cox proportional hazards model after adjustment for all relevant variables. **Results:** The SOT cohort had a significantly higher risk of DM than general patients (adjusted hazard ratio [aHR], 1.61; 95% confidence interval [CI], 1.51–1.72). Kidney and liver recipients, respectively, had DM incidence rates 1.57 (95% CI, 1.46–1.70) and 1.73 (95% CI, 1.53–1.94) times that of the general patients. **Conclusions:** SOT recipients had an elevated risk of DM. Among various organ recipients, liver recipients had the highest PTDM risk. Kidney and liver recipients demonstrated the highest DM risk at 6 months after their SOT. The risk of PTDM following an SOT may result in long-term consequences. Hence, we advise the critical need for proper management to mitigate related complications after transplantation.

## 1. Introduction

A solid organ transplant (SOT) is a therapeutic option for patients with chronic disease resulting from end-stage organ dysfunction and improves survival and quality of life [1,2]. In 2014, the International Expert Panel recommended a shift in terminology from new onset diabetes after transplantation (NODAT) back to posttransplant diabetes mellitus (PTDM) [3]. NODAT was used routinely before 2014 [4]. However, PTDM refers to the time of diagnosis rather than the time of occurrence. Specifically, PTDM refers to DM newly diagnosed after a transplant, irrespective of timing and whether DM was undetected prior to the transplant [3].

PTDM is a common complication following an SOT [5], with metabolic disturbances associated with various adverse outcomes that may increase the risks of morbidity and mortality [6]. PTDM is associated with a gradual decline in organ function, particularly in patients with longer survival periods [7,8]. The prevalence of PTDM after an SOT differs depending on the type of organ transplanted [9,10,11,12]. The incidence of PTDM following an SOT may also vary depending on the duration of the follow-up period [3,13]. Clinicians need a thorough understanding of the risk of PTDM following an SOT to effectively prevent relative complications. To address this, we conducted a retrospective cohort study using data from Taiwan’s National Health Insurance Research Database (NHIRD) between 2001 and 2018. This study aimed to examine the risk of PTDM across different types of SOTs and assess how the risk varies over different follow-up periods.

## 2. Materials and Methods

### 2.1. Data Sources

This study was a secondary analysis of data from the NHIRD on patients who underwent transplants at any point between 2001 and 2018. The NHIRD is a nationally representative database maintained by the Health and Welfare Data Science Center (HWDC) of the Ministry of Health and Welfare and includes detailed clinical records from both the inpatient and outpatient claims of the beneficiaries of Taiwan’s National Health Insurance program. This program has provided coverage for up to 99% of the country’s population since 1995. The NHIRD can serve as a foundation for the procurement of real-world evidence to support clinical decisions and health-care policy-making [14,15]. Diagnostic data within the NHIRD from before 2016 and from 2016 or later are, respectively, coded using International Classification of Diseases, Ninth Revision, Clinical Modification (ICD-9-CM) and International Classification of Diseases, Tenth Revision, Clinical Modification (ICD-10-CM) codes.

### 2.2. Ethics Statement

The work reported is in line with the STROCSS criteria [16]. Because the HWDC encrypts personally identifying data to protect the privacy of beneficiaries, this study was exempt from obtaining informed consent. This study protocol received an ethical review and approval by the Institutional Review Board of Chung Shan Medical University Hospital, Taiwan (No. CSMUH CS2-21134).

### 2.3. Study Participants

**Figure 1:**
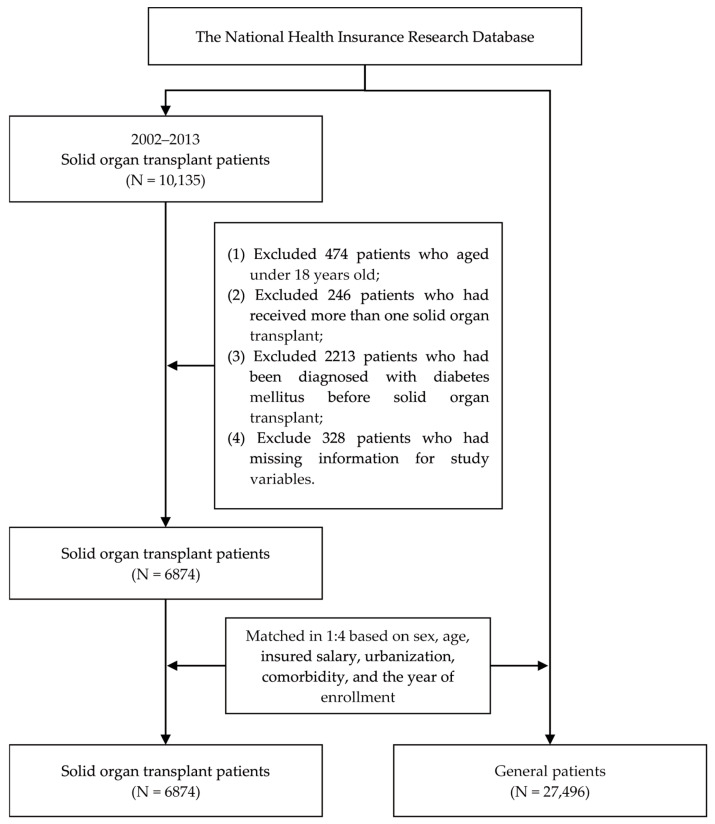
Flowchart of the patient selection process.

We enrolled patients aged over 18 years who underwent SOTs between 2002 and 2013, specifically, kidney (ICD-9-CM code V42.0), liver (ICD-9-CM code V42.7), or lung (ICD-9-CM code V42.6) transplants. Patients were excluded from our study if they received more than one SOT, had a DM diagnosis before their SOT, or had incomplete medical information in the NHIRD. This study used general patients without any DM diagnosis patients as the control group. To minimize potential selection bias resulting from the use of unbalanced covariates in observational studies, we employed 1:4 propensity score matching (PSM) to establish a matched cohort. PSM is a statistical matching technique that can be used to reduce potential confounding caused by unbalanced covariates in non-experimental settings. A propensity score is a probability that is calculated using a logistic regression model. The score is a unit of a certain characteristic assigned to a patient who received an SOT. These scores can help reduce or eliminate selection bias in observational studies by accounting for the characteristics of control individuals. We selected sex, age, insured salary, the urbanization level, Charlson’s comorbidity index (CCI), and the year of inclusion in the study as characteristics for matching. After matching, this study comprised 6874 patients who underwent an SOT and 27,496 matched general patients as the comparison. The patient selection process is presented in Figure 1.

### 2.4. Study Design

This retrospective cohort study investigated the risk of DM (ICD-9-CM code 250 or ICD-10-CM code E10–E14) by using data from the NHIRD of Taiwan. The date of an SOT was defined as the observation start date for SOT recipients, and the same date was assigned as the observation start date for corresponding members of the control group. To investigate the risk of DM, all participants were followed up until they were diagnosed with DM, died, or at the end of 2018, whichever occurred first. The comorbidities included in the analysis were hypertension (ICD-9-CM 401-405), hyperlipidemia (ICD-9-CM codes 272.0–272.4), obesity (ICD-9-CM code 287.0), rheumatoid arthritis (RA, ICD-9-CM code 714), inflammatory bowel disease (IBD, ICD-9-CM codes 555 and 556), hyperuricemia (ICD-9-CM code 790.6), cytomegalovirus (CMV, ICD-9-CM code 078.5), hepatitis B virus (HBV, ICD-9-CM codes 070.2 and 070.3), hepatitis C virus (HCV, ICD-9-CM codes 070.4, 070.5 and 070.70), sleep disturbance (ICD-9-CM code 780), and periodontitis (ICD-9-CM code 523).

### 2.5. Statistical Analysis

All statistical analyses in our study were conducted using SAS software version 9.4 (SAS Institute., Cary, NC, USA), with statistical significance indicated by *p* < 0.05. Chi-square tests were used to evaluate the distributions of baseline characteristics. In terms of multivariable analysis, given that our primary outcome (incident DM) is a time-to-event variable, a Cox proportional hazards model was employed to investigate the association between DM and undergoing an SOT after adjustment for all relevant variables. Results are presented as hazard ratios (HRs) with 95% confidence intervals (CIs). The sex, age, insured salary, urbanization, CCI, and comorbidities were the control variables to adjust the hazard ratio of the risk of incident DM. SOT recipients had an increased risk of DM at different periods after organ transplantation; thus, we conducted a sensitivity analysis at 6-month, 1-year, 3-year, and 5-year follow-ups [17]. A sensitivity analysis was also conducted to examine the risk of DM among SOT recipients after different follow-up periods, namely, 6 months and 1, 3, and 5 years.

## 3. Results

The mean follow-up period was 8.16 ± 3.41 years, and 5092 patients developed DM in 34,370 study individuals. Table 1 presents the baseline characteristics of the SOT recipients and matched control patients. Of the 34,370 study individuals, 19,732 were men and 14,638 were women. Of these patients, 6874 patients had undergone SOTs and 27,496 were matched controls. The average ages of the SOT recipients and matched controls were 47.52 ± 14.68 and 47.22 ± 11.73 years, respectively. Sex, age, insured salary, urbanization, and CCI did not differ significantly between the groups (*p* > 0.05). Among the 6874 SOT recipients, 3084 (44.86%) had hypertension, 874 (12.71%) had hyperlipidemia, 46 (0.67%) had RA, 28 (0.41%) had IBD, 201 had hyperuricemia (2.92%), 94 (1.37%) had CMV, 1493 (21.72%) had HBV, 663 (9.65%) had HCV, 1504 (21.88%) had sleep disturbance, and 44 (0.64%) had periodontitis. The distribution of each of the aforementioned comorbidities among the SOT cohort differed significantly from that of the matched cohort (*p* < 0.001).

Table 2 presents the risks of incident DM in the SOT and matched cohorts. In total, 5092 patients (14.82%) developed DM, 1443 patients (20.99%) of whom underwent SOTs. The DM incidence rates were 1.34 and 2.24 per 1000 person-years in the matched and SOT cohorts, respectively. In total, 2128 women (14.54%) and 2964 men (15.02%) developed DM; the incidence rates were 1.44 and 1.57 per 1000 person-years, respectively. Among the recipients of a kidney, liver, or lung, 956 (21.05%), 480 (21.09%), and 7 (12.28%), respectively, developed DM, for respective incidence rates of 2.12, 2.56, and 1.25 per 1000 person-years. Patients with hypertension, hyperuricemia, HCV, or sleep disturbances were more likely to develop DM than those without comorbidities.

With adjustments for confounding variables, the SOT cohort had a significantly higher risk of incident DM (adjusted HR [aHR], 1.61; 95% CI, 1.51–1.72) than the matched cohort. The incidence of DM among kidney and liver transplants was 1.57 times (95% CI, 1.46–1.70) and 1.73 (95% CI, 1.53–1.94) times that of the matched cohort, respectively. Patients with comorbid hypertension (aHR, 1.21; 95% CI, 1.13–1.28), hyperuricemia (aHR, 1.43; 95% CI, 1.20–1.71), HCV (aHR, 1.40; 95% CI, 1.24–1.58), or sleep disturbance (aHR, 1.10; 95% CI, 1.03–1.17) had a higher risk of incident DM after adjusting for relevant variables.

Table 3 presents the results of a sensitivity analysis conducted to investigate DM risk after various follow-up periods. Compared to the matched cohort, the SOT cohort had higher risks of incident DM after 6 months (aHR, 2.01; 95% CI, 1.67–2.42) and 1 (aHR, 1.83; 95% CI, 1.58–2.13), 3 (aHR, 1.94; 95% CI, 1.74–2.16), and 5 (aHR, 1.73; 95% CI, 1.58–1.90) years. Furthermore, kidney and liver recipients had higher risks of incident DM after 6 months and 1, 3, and 5 years.

## 4. Discussion

Large-scale epidemiological studies have investigated the risk of DM after an SOT. In this large population-based study, we found that SOT recipients had a higher risk of DM. Among the recipients of different organs, liver transplant recipients had the highest DM risk, that is, a 1.72-fold likelihood of developing DM. Renal transplant recipients had a 1.57-fold likelihood of developing DM. However, lung transplant recipients did not exhibit an elevated risk of DM. SOT recipients with comorbid hypertension, HCV, hyperuricemia, or sleep disturbances had a higher risk of DM than those without these comorbidities. Kidney and liver transplant recipients had the highest risks of DM at 6 months after their transplants; this heightened risk persisted, even after 1, 3, and 5 years.

In the current study, people with SOTs had a higher risk of DM than matched controls. The pathogenesis of PTDM is multifactorial. The pathogenesis of PTDM mainly involves pancreatic β-cell dysfunction and insulin resistance [18]. However, a study indicated that β-cell dysfunction alone is the major factor contributing to DM in kidney recipients [19]. The pathogenesis of PTDM is similar to that of type 2 DM, sharing characteristics such as insulin resistance, hypertriglyceridemia, obesity, decompensated insulin release, hypertension, and low-grade inflammation [20,21]. However, the underlying mechanisms of the two conditions may differ [22]. Nevertheless, impairments in insulin-mediated glucose uptake in peripheral tissue [23,24], insulin-mediated suppression of hepatic glucose output [24], insulin release [25], and the incretin axis between the gut and pancreas [26] (reinforcing the impairment of β-cell function and enhancing glucagon production) are common to both T2DM and PTDM. Increased renal gluconeogenesis and proximal tubular sodium–glucose reabsorption are present in T2DM [27] but have not been observed in PTDM

In addition to the transplanted organ, factors that may affect the incidence of PTDM include age, race, body mass index, and immunosuppression regimen [22]. PTDM is associated with long-term graft function impairment, decreased graft survival, increased risks of cardiovascular disease and infection, and acute graft rejection [7,8].

Compared with cyclosporine A, tacrolimus can improve graft survival and prevent acute rejection after transplantation, but it contributes to an increase in the occurrence of PTDM [28,29]. Therefore, as the most commonly used immunosuppressants after transplantation, blood glucose concentrations should be closely monitored in recipients taking tacrolimus [28,30]. However, we did not analyze the relationship between immunosuppressive drugs and the occurrence of PTDM in SOT recipients.

The current study discovered that, among the recipients of different organs, liver recipients had the highest risk of PTDM, specifically a 1.72-fold likelihood of DM; prior studies have indicated that PTDM occurs in 12–45% of liver recipients [3,31,32]. Another study indicated that PTDM is a significant causal factor of morbidity and mortality in liver recipients [33]. Liver transplant recipients with sustained PTDM had significantly higher risks of death due to infection, graft failure resulting from chronic graft rejection, and late-onset thrombosis of the hepatic artery [34].

The current study found that kidney recipients had a 1.57-fold likelihood of developing DM. One study found that DM incidence after renal transplants ranges between 7.9% and 50% [35]; however, another study found that DM incidence after such transplants ranges between 10% and 20% [22]. PTDM has been associated with an increased mortality, which is well documented in recipients who have received kidney and heart transplants [22,36]. Furthermore, PTDM has been associated with decreased graft survival [37,38], premature cardiovascular disease, and death in renal transplant recipients [39].

The current study found no association between lung transplants and PTDM. Previous study indicated that DM is a significant concern for lung transplant recipients. Pre-existing DM diagnosed before lung transplantation adversely impacts posttransplant survival in patients with cystic fibrosis. Patients with cystic fibrosis and pre-existing DM experience reduced survival rates compared to those with cystic fibrosis who do not have pre-existing DM [40].

The risk factors for PTDM are complex and associated with numerous variables [18]. PTDM is more likely in patients with pre-existing risk factors for T2DM, including older age, male sex, a family history of T2DM, a high-risk ethnicity, and obesity [1,41]. In the current study, people with SOTs and aged 51–60 years had the highest DM risk. Additionally, male patients had a higher risk than female patients. PTDM is a common complication in lung transplant recipients taking tacrolimus-based immunosuppression [42].

Age is a major risk factor for PTDM. With advancing age, islet β cells age and undergo apoptosis, resulting in decreased insulin secretion, increased insulin resistance, and a 2.2-fold increase in PTDM risk for SOT recipients older than 45 years [22]. Another study indicated that an age of >55 years is a significant risk factor for PTDM [43]. Furthermore, older age is independently correlated with a higher risk of irreversible PTDM [18].

The current study also found that patients with higher CCIs, particularly those >2, had a higher PTDM risk. Age and CCI are the two primary risk factors for PTDM across all time periods [44]. Moreover, PTDM is more prevalent among male SOT recipients than among female SOT recipients [45].

The current study observed that SOT recipients with comorbid hypertension, HCV, hyperuricemia, or sleep disturbances had a higher PTDM risk than those without these comorbidities. Risk factors for PTDM, including insulin resistance, hypertension, hypertriglyceridemia, and obesity, are similar to those for T2DM, but PTDM is influenced by specific predisposing posttransplant factors [36]. Hypertension is a risk factor for DM after renal transplants [46]. High serum uric acid levels are associated with a higher PTDM risk in kidney recipients, independently of metabolic syndrome components and transplant-related variables, including immunosuppressive therapy, HCV, and CMV [47]. HCV is a PTDM risk factor [48] and has been associated with a 2.5- to 4-fold relative risk of PTDM after liver transplants [9,49,50,51]. HCV can induce posttransplant insulin resistance [52]. Several meta-analyses have indicated that patients with HCV have higher risks of mortality and graft loss after renal transplants than those without transplants [53,54]. CMV is another comorbidity associated with PTDM. Because of immunosuppression, CMV infection is a common complication of transplants [36]. CMV infection may also play a role in the pathogenesis of PTDM because it is associated with an increased PTDM risk [55]. CMV induces the secretion of proinflammatory cytokines and the destruction of pancreatic β cells [1]. However, we found that SOT recipients with comorbid CMV infection did not have a higher risk for PTDM. A systematic review and meta-analysis identified sleep disturbances as a risk factor for DM [56].

The current study discovered that kidney and liver recipients had the highest PTDM risk 6 months after their transplants, a risk that remained high even after 1, 3, and 5 years. The 2014 international consensus meeting on PTDM identified two critical periods for assessment: 46–365 days and >365 days after the procedure [3]. The incidence of PTDM follows a biphasic pattern, with the first peak occurring in the first few months and the second over the subsequent 2–3 years [13]. One study observed the highest PTDM incidence at 46–365 days after the SOT [44]. In addition, age and CCI were the two primary risk factors for PTDM for all investigated periods [44]. Another study observed the highest PTDM incidence within 6 months after the procedure [57]. Furthermore, the incidence of PTDM was discovered to be 20.5% at 6 months after renal transplants [58]. A Taiwanese population-based study indicated that the PTDM incidence was the highest within the first year after a renal transplant [59]. Among lung transplant recipients, 33% had PTDM after 3 months, 30% had PTDM after 1 year, and 24% had PTDM after 2 years [22]. However, the decline in PTDM could indicate a competing risk estimate, considering more recipients with PTDM died during the first year [22].

The current study suggests that patients with PTDM require regular glucose monitoring and the early intervention of the endocrinologist with timely treatment of hyperglycemia to improve long-term outcomes. An awareness and identification of PTDM are important. Thus, understanding PTDM would help in reducing severe complications and minimizing its impact following transplantation.

The present study has several strengths. First, it employed a nationwide population-based study design. Patients were selected from the entire population of Taiwan and followed for extended periods, thereby yielding a large, representative sample with high statistical utility. The population-based design may have minimized selection bias, which is common in observational studies. Second, we investigated the risk of DM in SOT recipients at different time points after their transplants at 6 months and 1, 3, and 5 years.

The current study also had limitations. First, some inherent flaws may be associated with retrospective cohort analysis, such as selective reporting bias and data errors. Second, we performed a retrospective cohort study using the NHIRD, which is a secondary database. The NHIRD does not include laboratory or medical examination data; thus, we could not assess diagnostic criteria per the consensus guidelines. Third, smoking habits, alcohol consumption, body mass index, HbA1c levels, personal history, physical activity, and dietary habits are associated with PTDM risk, but related data could not be accessed for this study. Although information such as smoking habits and alcohol consumption cannot be obtained from the NHIRD, this study includes the total population of the nation; thus, this large sample size study was representative, which may increase statistical precision. There is no doubt about the representativeness of this study. Fourth, all diagnoses in this study were based on ICD-9-CM or ICD-10-CM codes. However, the Bureau of the National Health Insurance in Taiwan randomly reviews charts and conducts patient interviews to ensure diagnostic accuracy, which bolsters the validity and accuracy of the analyzed NHIRD data. We acknowledge the possibility that we did not control for some confounding variables.

## 5. Conclusions

This study provides large-scale, population-based, longitudinal evidence that SOT recipients experience an elevated DM risk. Among the recipients of different organs, the liver recipients had the highest PTDM risk. The risk of PTDM following an SOT could have long-term consequences. Therefore, we emphasize the importance of effective management DM to reduce associated complications following transplantation.

## Figures and Tables

**Table 1:** Baseline characteristics of the study subjects after matching.

Variables	General Patients		SOT Patients		*p*-Value
	N	%	N	%	
Total	27,496	100.00	6874	100.00	
Sex ^1^					0.965
Female	11,712	42.60	2926	42.57	
Male	15,784	57.40	3948	57.43	
Age (year) ^1^					0.999
≤40	7591	27.61	1893	27.54	
41–50	8085	29.40	2027	29.49	
51–60	8516	30.97	2127	30.94	
≥61	3304	12.02	827	12.03	
Mean ± SD	47.52 ± 14.68		47.22 ± 11.73		
Insured salary (NTD) ^1,2^					0.995
≤21,000	13,580	49.39	3399	49.45	
21,001–33,000	5901	21.46	1475	21.46	
≥33,001	8015	29.15	2000	29.10	
Urbanization ^1,2^					0.997
Level 1	8160	29.68	2053	29.87	
Level 2	9040	32.88	2266	32.96	
Level 3	4551	16.55	1138	16.56	
Level 4	3624	13.18	900	13.09	
Level 5	333	1.21	83	1.21	
Level 6	828	3.01	205	2.98	
Level 7	960	3.49	229	3.33	
CCI score ^1,3^					0.998
0	1276	4.64	322	4.68	
1	1040	3.78	258	3.75	
2	7596	27.63	1898	27.61	
≥3	17,584	63.95	4396	63.95	
Enrolled year ^1^					1.000
2002	1376	5.00	344	5.00	
2003	1576	5.73	394	5.73	
2004	2148	7.81	537	7.81	
2005	2772	10.08	693	10.08	
2006	3044	11.07	761	11.07	
2007	2516	9.15	629	9.15	
2008	2760	10.04	690	10.04	
2009	2280	8.29	570	8.29	
2010	2224	8.09	556	8.09	
2011	2268	8.25	567	8.25	
2012	2152	7.83	538	7.83	
2013	2380	8.66	595	8.66	
Comorbidities					
Hypertension					<0.001
No	18,890	68.70	3790	55.14	
Yes	8606	31.30	3084	44.86	
Hyperlipidemia					<0.001
No	20,828	75.75	6000	87.29	
Yes	6668	24.25	874	12.71	
Obesity					<0.001
No	27,261	99.15	6867	99.90	
Yes	235	0.85	7	0.10	
RA ^3^					<0.001
No	26,913	97.88	6828	99.33	
Yes	583	2.12	46	0.67	
IBD ^3^					0.002
No	27,286	99.24	6846	99.59	
Yes	210	0.76	28	0.41	
Hyperuricemia					<0.001
No	27,177	98.84	6673	97.08	
Yes	319	1.16	201	2.92	
Cytomegalovirus					<0.001
No	27,488	99.97	6780	98.63	
Yes	8	0.03	94	1.37	
HBV ^3^					<0.001
No	26,165	95.16	5381	78.28	
Yes	1331	4.84	1493	21.72	
HCV ^3^					<0.001
No	26,883	97.77	6211	90.35	
Yes	613	2.23	663	9.65	
Sleep disturbance					<0.001
No	19,927	72.47	5370	78.12	
Yes	7569	27.53	1504	21.88	
Periodontitis					0.015
No	27,235	99.05	6830	99.36	
Yes	261	0.95	44	0.64	

^1^ Variables for propensity score matching. ^2^ NTD is New Taiwan Dollar (NTD 1 ≈ USD 0.034); urbanization level 1 denoted the highest degree of urbanization, whereas level 7 denoted the lowest degree of urbanization. ^3^ Abbreviations: CCI, Charlson’s comorbidity index; RA, rheumatoid arthritis; IBD, inflammatory bowel disease; HBV, hepatitis B virus; HCV, hepatitis C virus.

**Table 2:** The multivariable analysis of the risk of incident diabetes mellitus via a Cox proportional hazard model.

Variables	Incident Diabetes Mellitus									
	No		Yes		IR ^1^	Adjusted Model				
	N	%	N	%		HR	95% CI			*p*-Value
Total	29,278	85.18	5092	14.82	1.51					
Patients cohort										
General (ref.)	23,847	86.73	3649	13.27	1.34	1				
SOT	5431	79.01	1443	20.99	2.24	1.61	1.51	-	1.72	<0.001
Kidney	3585	78.95	956	21.05	2.12	1.57	1.46	-	1.70	<0.001
Liver	1796	78.91	480	21.09	2.56	1.73	1.53	-	1.94	<0.001
Lung	50	87.72	7	12.28	1.25	0.99	0.47	-	2.07	0.969
Sex										
Female (ref.)	12,510	85.46	2128	14.54	1.44	1				
Male	16,768	84.98	2964	15.02	1.57	1.08	1.02	-	1.15	0.006
Age (year)										
≤40 (ref.)	8564	90.30	920	9.70	0.92	1				
41–50	8506	84.12	1606	15.88	1.58	1.67	1.54	-	1.81	<0.001
51–60	8768	82.38	1875	17.62	1.92	1.95	1.79	-	2.11	<0.001
≥61	3440	83.27	691	16.73	1.87	1.82	1.64	-	2.02	<0.001
Insured salary (NTD) ^2^	46.97 ± 14.52		50.24 ± 11.36							
≤21,000 (ref.)	14,327	84.38	2652	15.62	1.50	1				
21,001–33,000	6375	86.43	1001	13.57	1.57	0.98	0.91	-	1.06	0.609
≥33,001	8576	85.63	1439	14.37	1.50	0.95	0.89	-	1.01	0.123
Urbanization ^2^										
Level 1 (ref.)	8768	85.85	1445	14.15	1.41	1				
Level 2	9621	85.10	1685	14.90	1.52	1.06	0.98	-	1.13	0.130
Level 3	4845	85.16	844	14.84	1.56	1.11	1.02	-	1.20	0.021
Level 4	3824	84.53	700	15.47	1.61	1.12	1.02	-	1.22	0.019
Level 5	347	83.41	69	16.59	1.77	1.27	1.00	-	1.62	0.055
Level 6	869	84.12	164	15.88	1.67	1.10	0.93	-	1.29	0.275
Level 7	1004	84.44	185	15.56	1.68	1.13	0.97	-	1.31	0.130
CCI score ^3^										
0 (ref.)	1431	89.55	167	10.45	0.98	1				
1	1087	83.74	211	16.26	1.51	1.41	1.15	-	1.73	<0.001
2	8024	84.52	1470	15.48	1.53	1.42	1.21	-	1.67	<0.001
≥3	18,736	85.24	3244	14.76	1.55	1.27	1.08	-	1.49	0.003
Comorbidities										
Hypertension										
No (ref.)	19,668	86.72	3012	13.28	1.32					
Yes	9610	82.21	2080	17.79	1.93	1.21	1.13	-	1.28	<0.001
Hyperlipidemia										
No (ref.)	22,882	85.29	3946	14.71	1.48					
Yes	6396	84.81	1146	15.19	1.66	1.01	0.94	-	1.08	0.873
Obesity										
No (ref.)	29,073	85.19	5055	14.81	1.51					
Yes	205	84.71	37	15.29	1.68	1.22	0.88	-	1.69	0.225
RA ^3^										
No (ref.)	28,759	85.23	4982	14.77	1.51					
Yes	519	82.51	110	17.49	1.75	1.18	0.97	-	1.42	0.094
IBD ^3^										
No (ref.)	29,073	85.18	5059	14.82	1.52					
Yes	205	86.13	33	13.87	1.43	0.96	0.68	-	1.35	0.818
Hyperuricemia										
No (ref.)	28,884	85.33	4966	14.67	1.50					
Yes	394	75.77	126	24.23	2.71	1.43	1.20	-	1.71	<0.001
Cytomegalovirus										
No (ref.)	29,192	85.19	5076	14.81	1.51					
Yes	86	84.31	16	15.69	1.63	0.78	0.48	-	1.28	0.328
HBV ^3^										
No (ref.)	26,923	85.35	4623	14.65	1.49					
Yes	2355	83.39	469	16.61	1.87	0.99	0.89	-	1.09	0.778
HCV ^3^										
No (ref.)	28,298	85.51	4796	14.49	1.47					
Yes	980	76.80	296	23.20	2.72	1.40	1.24	-	1.58	<0.001
Sleep disturbance										
No (ref.)	21,667	85.65	3630	14.35	1.45					
Yes	7611	83.89	1462	16.11	1.72	1.10	1.03	-	1.17	0.004
Periodontitis										
No (ref.)	29,024	85.20	5041	14.80	1.51					
Yes	254	83.28	51	16.72	1.92	1.16	0.88	-	1.53	0.29

^1^ IR, incident rate per 1000 person-year. ^2^ NTD is New Taiwan Dollar (NTD 1 ≈ USD 0.034); urbanization level 1 denoted the highest degree of urbanization, whereas level 7 denoted the lowest degree of urbanization. ^3^ Abbreviations: CCI, Charlson’s comorbidity index; RA, rheumatoid arthritis; IBD, inflammatory bowel disease; HBV, hepatitis B virus; HCV, hepatitis C virus.

**Table 3:** Sensitivity analysis to investigate the risk of incident diabetes mellitus at different follow-up periods.

Variables	SOT Patients vs. General Patients (Ref.) ^1^			
	6 Month	1 Year	3 Year	5 Year
	aHR (95% CI)	aHR (95% CI)	aHR (95% CI)	aHR (95% CI)
SOT	2.01 (1.67–2.42)	1.83 (1.58–2.13)	1.94 (1.74–2.16)	1.73 (1.58–1.90)
Kidney	1.91 (1.53–2.37)	1.65 (1.37–1.97)	1.78 (1.57–2.02)	1.67 (1.50–1.86)
Liver	2.33 (1.71–3.18)	2.40 (1.87–3.07)	2.39 (1.98–2.87)	1.91 (1.63–2.24)
Lung	-	-	1.77 (0.63–4.94)	1.09 (0.39–3.04)

^1^ All models were analyzed using the Cox proportional hazards model. Extraneous factors adjusted in the model were sex, age, insured salary, urbanization, CCI, and comorbidities.

## Data Availability

The database used to support the findings of this study was provided by the Health and Welfare Data Science Center, Ministry of Health and Welfare (HWDC, MOHW) under license and cannot be made freely available. Requests for access to these data should be made to HWDC (https://dep.mohw.gov.tw/dos/cp-5119-59201-113.html, accessed on 20 October 2024).
